# Fucoidan from *Laminaria japonica* Inhibits Expression of GLUT9 and URAT1 via PI3K/Akt, JNK and NF-κB Pathways in Uric Acid-Exposed HK-2 Cells

**DOI:** 10.3390/md19050238

**Published:** 2021-04-23

**Authors:** Yu Zhang, Xiaohui Tan, Zhen Lin, Fangping Li, Chunyan Yang, Haiying Zheng, Lingyu Li, Huazhong Liu, Jianghua Shang

**Affiliations:** 1 College of Chemistry and Environmental Science, Guangdong Ocean University, Zhanjiang 524088, China; joanne96zy@163.com (Y.Z.); tanxiaohuiii@163.com (X.T.); linz199771@163.com (Z.L.); lifangping1993@163.com (F.L.); 2 Guangxi Key Laboratory of Buffalo Genetics, Reproduction and Breeding, Guangxi Buffalo Research Institute, Chinese Academy of Agricultural Sciences, Nanning 530001, China; ycy3106@163.com (C.Y.); haiyingzheng@126.com (H.Z.); lly01202020@163.com (L.L.)

**Keywords:** fucoidan, uric acid, urate transporter 1, glucose transporter 9

## Abstract

This work aimed to investigate the effect of fucoidan (FPS) on urate transporters induced by uric acid (UA). The results showed that UA stimulated the expression of glucose transporter 9 (GLUT9) and urate transporter 1 (URAT1) in HK-2 cells, and FPS could reverse the effect. Moreover, UA could activate NF-κB, JNK and PI3K/Akt pathways, but both pathway inhibitors and FPS inhibited the UA-induced activation of these three pathways. These data suggested that FPS effectively inhibited the expression induction of reabsorption transporters URAT1 and GLUT9 by UA, through repressing the activation of NF-κB, JNK and PI3K/Akt signal pathways in HK-2 cells. The in vitro research findings support the in vivo results that FPS reduces serum uric acid content in hyperuricemia mice and rats through inhibiting the expression of URAT1 and GLUT9 in renal tubular epithelial cells. This study provides a theoretical basis for the application of FPS in the treatment of hyperuricemia.

## 1. Introduction

Hyperuricemia is considered a metabolic disease that induces gout, chronic nephrosis and insulin resistance. Hyperuricemia accelerates vasculopathy and the occurrence and development of abnormal glucose tolerance, and is closely related to hypertension, atherosis, coronary heart disease, lipid metabolism disorder, obesity and sexual dysfunction [1,2]. An epidemiologic study revealed that hyperuricemia incidence showed an increasing and younger trend. The incidence rate was 13%; incidence in males (18.5%) was higher than in females (8%) in China [2]. Hyperuricemia pathogenesis includes excessive production and declined excretion. A background study showed that 90% of primary hyperuricemia cases resulted from declined excretion of urate from the kidneys [3]. In total, 70% of serum uric acid is excreted from the kidneys [4], but 90–95% of uric acid can be reabsorbed into the blood [5]. So, regulating the function of renal tubular urate transporters and inhibiting the reabsorption of urate is critical for hyperuricemia treatment.

Urate anion transporter 1 (URAT1) and glucose transporter 9 (GLUT9) are two important transporters for urate reabsorption. URAT1 mediates extracellular urate transport into cells; in renal tubular epithelial cells, URAT1-mediated uptake of urate from the lumen is the first step in urate reabsorption [6]. GLUT9 is the only reabsorption-associated protein located on the side of the basal membrane of renal tubular epithelial cells, and is the last step of urate reabsorption. Functional collaboration between URAT1 on the apical membrane and basolateral GLUT9 is essential for renal reabsorption of urate in human kidneys [6]. The two transporters are targets of present uricosuric drugs.

Drugs for hyperuricemia have been classified into two categories; uricostatic drugs such as allopurinol, febuxostat, topiroxostat and BCX-4208, and uricosuric drugs including probenecid, benzbromarone, sulphinpyrazone, pyrazinamide, RDEA-594 and RDEA-684. However, these clinical drugs have an unbearable, untoward effect on patients [7,8,9]. So, screening nontoxic agents for hyperuricemia treatment is urgent, and Chinese traditional medicines have been considered to be good options [10,11].

Fucoidan (FPS) is a kind of sulfated and heterogeneous polysaccharide which originates from brown seaweeds. FPS is rich in fucose and sulfate groups, and has many bioactive properties such as antitumor, antivirus, antioxidation, antithrombotic, anticoagulant, anti-inflammatory and immunomodulatory. It also has a clinical application in the treatment of renal, hepatic and uropathic disorders [12,13]. FPS is mainly distributed in the kidneys, is highly effective in treating various diseases, and has been used as clinical drug for renal diseases from 2003 in China [14,15]. In 2013, it was first reported that FPS inhibited URAT1 expression; consequently, it can decline serum uric acid in hyperuricemia rats [16]. Afterwards, our previous work found that FPS not only inhibited the expression of URAT1, but also suppressed activities of xanthine oxidase and adenosine deaminase, resulting in the decrease in serum uric acid in hyperuricemia mice [17,18]. In 2019, Chau and co-workers reconfirmed that FPS inhibited the activity of xanthine oxidase and the expression of URAT1 and GLUT9 in hyperuricemia rats [19]. These reports suggest that FPS is effective in the treatment of hyperuricemia by repressing the production of uric acid and promoting the excretion of uric acid from the kidneys. However, it is still unknown what mechanism, involved in the regulation of the FPS-inhibited expression of URAT1 and GLUT9 in renal tubular epithelial cells, which will be unraveled in this study.

## 2. Results

### 2.1. FPS Inhibits UA-Induced Expression of URAT1 and GLUT9 in HK-2 Cells

**Figure 1:**
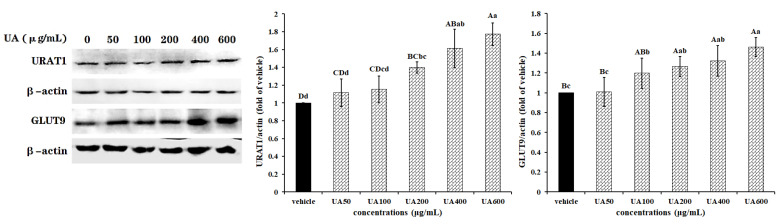
UA-promoted expression of URAT1 and GLUT9 in HK-2 cells. Cells of 80% confluency were exposed to different concentrations of UA (0, 50,100, 200, 400 and 600 μg/mL, Sigma) for 24 h, and were then lysed in radio immunoprecipitation assay (RIPA) lysis buffer supplemented with phenylmethanesulfonyl fluoride (PMSF). Western blotting analysis was used to determine protein contents of URAT1 and GLUT9, *β*-actin was used to normalize them. Data were expressed as folds of vehicle. ^ABCD^ *p* < 0.01, ^abcd^ *p* < 0.05.

**Figure 2:**
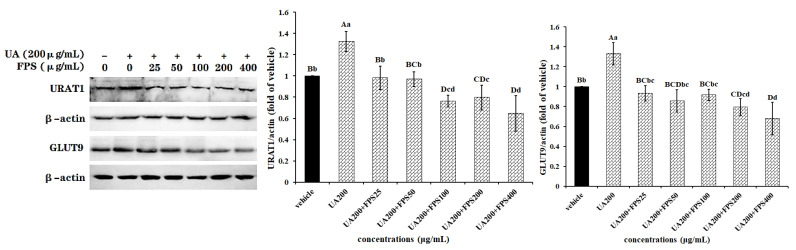
FPS repressed the UA-induced expression of URAT1 and GLUT9 in HK-2 cells. Cells were grown to 80% confluence, and were then treated with combinations of 200 μg/mL UA and different concentrations of FPS (0, 25, 50, 100, 200 and 400 μg/mL) for 24 h. Cells were lysed in RIPA buffer supplemented with PMSF. URAT1 and GLUT9 expression levels were assessed using Western blotting. Data were normalized with *β*-actin content, and were expressed as folds of vehicle. ^ABCD^ *p* < 0.01, ^abcd^ *p* < 0.05.

The data presented in Figure 1 show that the UA-induced expression of URAT1 and GLUT9 in HK-2 cells occurred in a dose-dependent fashion. At a dose of 200 μg/mL, UA markedly increased the expression of GLUT9 and URAT1 proteins (*p* < 0.01), and the protein contents were positively associated with UA dosage. It was also found that a low dosage of UA failed to modify the expression level of transporter URAT1 (100 μg/mL, *p* > 0.05) and GLUT9 (50 μg/mL, *p* > 0.05), which suggests that urate at a low concentration may not affect the renal function of urate excretion, but a high concentration does. Namely, a high urate level may inhibit urate excretion from the kidneys in hyperuricemia patients, resulting in a higher urate content. However, the UA-induced expression of the two reabsorption transporters was significantly inhibited by the natural marine polysaccharide fucoidan at a very low dosage of 25 μg/mL (*p* < 0.01). Moreover, along with an increase in FPS concentration, protein contents of URAT1 and GLUT9 became lower and lower, and more significant differences were observed. Interestingly, FPS (100 μg/mL or 200 μg/mL) significantly decreased the expression level of URAT1 or GLUT9 to below the normal level (*p* < 0.01) (Figure 2). These data indicate that FPS may highly effectively promote urate excretion from the kidneys in hyperuricemia cases. Based on the above data, 200 μg/mL of UA and 25 μg/mL of FPS were selected for the following work.

### 2.2. FPS Inhibits UA-Induced Expression of URAT1 and GLUT9 via Repressing NF-κB Pathway

**Figure 3:**
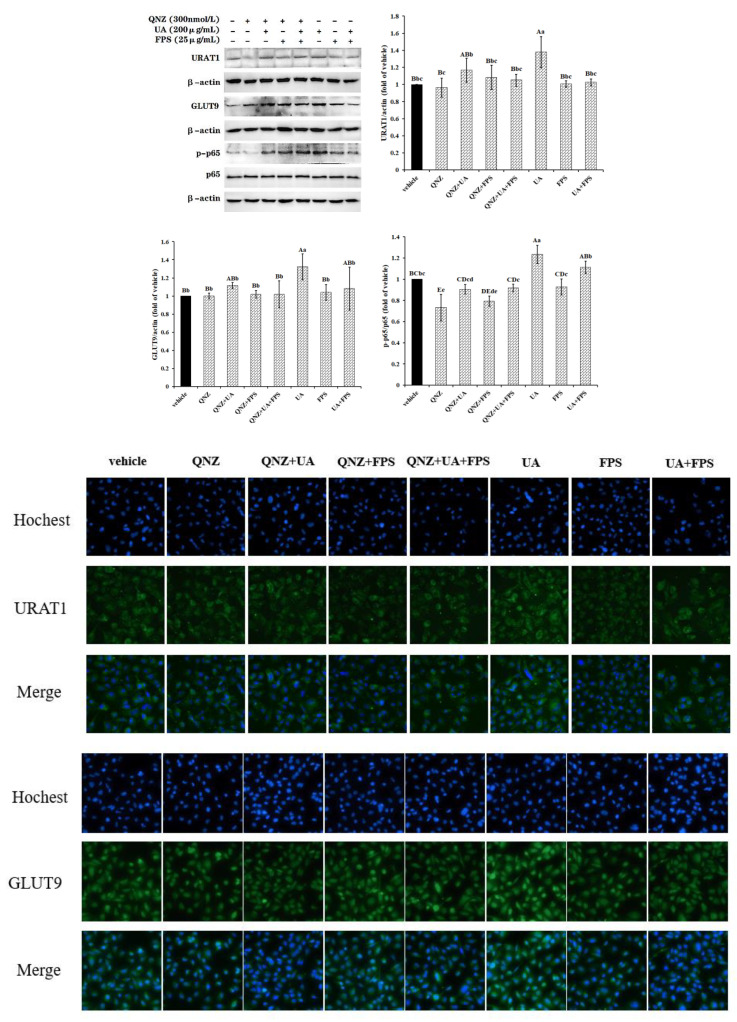
FPS repressed the UA-induced expression of URAT1 and GLUT9 via NF-κB signal pathway in HK-2 cells. Cells (80% confluence) were exposed to NF-κB pathway inhibitor QNZ (300 nmol/L) for 2 h and then to UA (200 μg/mL) and FPS (25 μg/mL) for another 24 h. Cell lysate in RIPA buffer containing PMSF were used to determine URAT1 and GLUT9 contents normalized with *β*-actin, and *p*-p65 content normalized with p65. Data were expressed as folds of vehicle. ^ABCDE^ *p* < 0.01, ^abcde^ *p* < 0.05.

Figure 3 shows that NF-κB pathway inhibitor EVP4593 (QNZ) successfully inhibited activation of p65 protein (*p* < 0.01) but did not affect the expression of URAT1 and GLUT9 (*p* > 0.05), suggesting that QNZ could not change the urate excretion function of control renal tubular epithelial cells. However, for the cells exposed to a high dosage of UA, QNZ sharply blocked p65 protein phosphorylation caused by UA (*p* < 0.01), maintained the normal activation level (*p* > 0.05 vs. vehicle), and kept contents of GLUT9 and URAT1 in UA-exposed cells at levels of insignificant difference compared to vehicle-treated cells (*p* > 0.05). Data indicated that UA induced the expression of GLUT9 and URAT1 via activating the NF-κB signal pathway.

Compared with UA-treated cells, cells co-treated with FPS and UA were observed to have significantly reduced levels of phospho-p65 (*p* < 0.05), GLUT9 (*p* < 0.05) and URAT1 (*p* < 0.01). This reduction was almost the same as with vehicle cells (*p* > 0.05), indicating that the NF-κB signal pathway participated in the regulatory mechanisms of FPS, inhibiting the UA-induced expression of URAT1 and GLUT9 in HK-2 cells. It was also found that QNZ effectively promoted the inhibition of FPS towards UA-induced p65 phosphorylation (*p* < 0.01 vs. UA + FPS), but did not modify the inhibition of FPS on the UA-induced expression of URAT1 and GLUT9 (*p* > 0.05 vs. vehicle).

Additionally, as for control cells, FPS had no effect on both the expression of URAT1 and GLUT9 and the phosphorylation of p65 protein (*p* > 0.05), indicating that FPS could not change the expression level of URAT1 and GLUT9, and the activation level of the NF-κB signal pathway. The signal pathway inhibitor, QNZ, did not modify protein contents of GLUT9 and URAT1 in FPS-exposed cells, but obviously declined the content of p65 in the cells (*p* < 0.01).

### 2.3. JNK Pathway Involved in FPS Suppressing UA-Induced Expression of URAT1 and GLUT9

**Figure 4:**
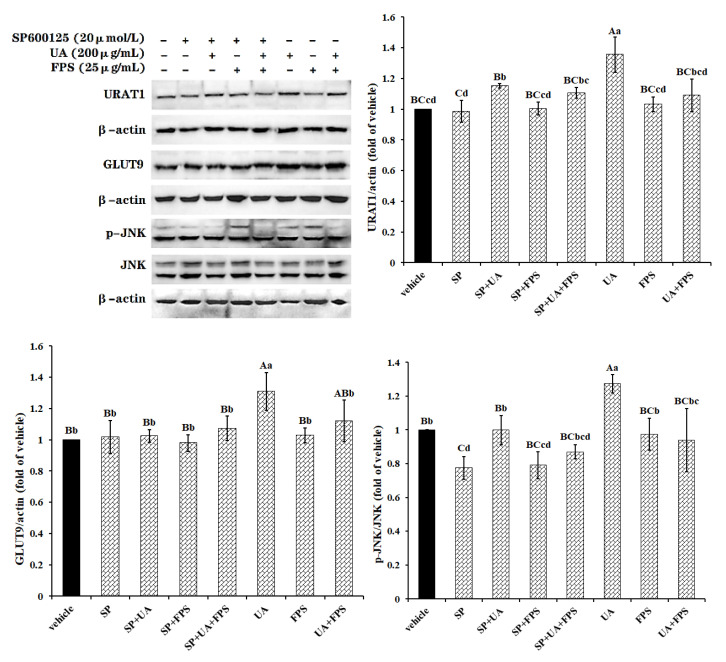

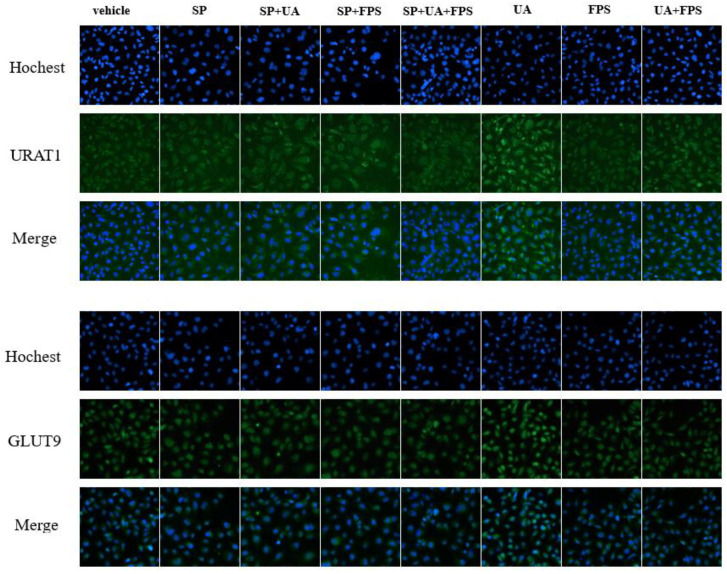
FPS repressed the UA-induced expression of URAT1 and GLUT9 via the JNK signal pathway in HK-2 cells. Cells at 80% confluence were treated with JNK pathway inhibitor SP600125 (SP, 20 μmol/L) for 2 h, and were then exposed to UA (200 μg/mL) and FPS (25 μg/mL) for another 24 h. Cell lysate in RIPA buffer containing PMSF were conducted to determine relative contents of URAT1, GLUT9 and *p*-JNK. Data were expressed as folds of vehicle. ^ABC^ *p* < 0.01, ^abcd^ *p* < 0.05.

As shown in Figure 4, both JNK signal pathway inhibitor SP600125 and FPS suppressed the expression induction of URAT1 and GLUT9 by UA (*p* < 0.05 or *p* < 0.01); contents of the two transporters in the UA + FPS and SP + UA treated cells were approximately equal to that of vehicle cells (*p* > 0.05). Meanwhile, both SP600125 and FPS clearly inhibited the UA-induced phosphorylation of JNK protein (*p* < 0.01). FPS did not change the expression of URAT1 and GLUT9, and the activation of JNK protein in control cells (*p* > 0.05). These results suggest that FPS inhibited the expression induction of urate reabsorption transporters through suppressing the activation of JNK by UA. In addition, inhibitor SP600125 did not change the contents of the two transporters (*p* > 0.05), but declined phospho-JNK protein content (*p* < 0.05). It could be concluded from the data in Figure 4 that FPS blocked the activation of the JNK signal pathway induced by UA, resulting in the inhibition of the expression induction of URAT1 and GLUT9 by UA.

Additionally, both the inhibitor and FPS maintained the expression level of URAT1 and GLUT9 and the phosphorylation of signal molecules JNK appeared to be at a normal level in UA-exposed cells. However, it was different that the inhibitor significantly declined the activation level of the signal pathway in control cells (*p* < 0.01), but FPS did not (*p* > 0.05).

### 2.4. FPS Inhibits Expression of GLUT9 and URAT1 through Repressing UA-Induced Activation of PI3K/Akt Pathway in HK-2 Cells

**Figure 5:**
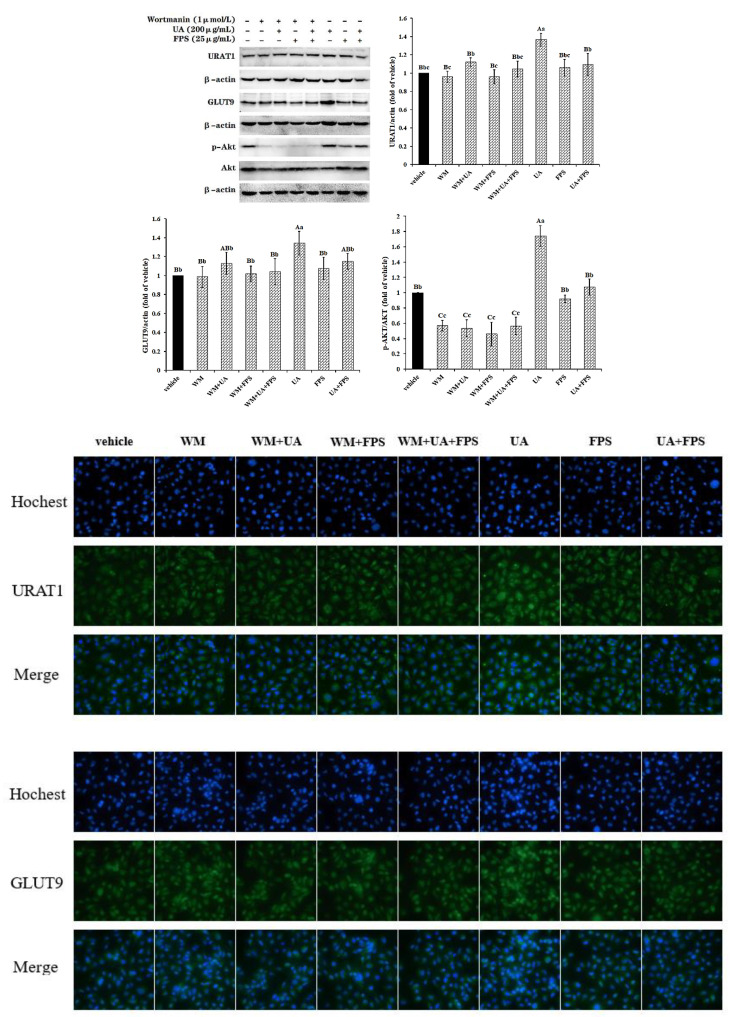
FPS-repressed UA-induced expression of URAT1 and GLUT9 via PI3K/Akt signal pathway in HK-2 cells. Cells were grown to 80% confluence, treated with PI3K/Akt pathway inhibitor wortmanin (WM, 1 μmol/L) for 2 h, and were then exposed to UA (200 μg/mL) and FPS (25 μg/mL) for 24 h. Cell lysate in RIPA buffer containing PMSF were used to determine relative contents of URAT1, GLUT9 and p-AKT proteins. Data were expressed as folds of vehicle. ^ABC^ *p* < 0.01, ^abc^ *p* < 0.05.

Data are presented in Figure 5. Although both FPS and wortmannin did not change the expression patterns of URAT1 and GLUT9 in control cells, they effectively declined the high levels of the two transporters in UA-exposed cells to normal levels (*p* < 0.05 or *p* < 0.01). Additionally, FPS exposure blocked the activation of the PI3K/Akt pathway caused by UA (*p* < 0.01). Similarly to SP600125, wortmannin failed to reduce contents of URAT1 and GLUT9 to below normal levels in untreated cells, but decreased the activated signal molecule p-AKT in untreated cells and in UA + FPS treated cells to below normal content (*p* < 0.01). Additionally, wortmannin declined the activation level of the PI3K/Akt signal pathway in control cells (*p* < 0.01), but FPS did not (*p* > 0.05).

## 3. Discussion

The transport processes of UA in the kidneys mainly includes glomerular filtration, renal tubular reabsorption and secretion, and post-secretion reabsorption, etc. Except glomerular filtration, the other processes require the participation of urate transporter protein. URAT1 is a transporter in proximal convoluted tubules and is responsible for UA reabsorption from renal lumen into epithelial cells; GLUT9 successfully transports UA from epithelial cells into blood [4]. GLUT9 accelerates UA reabsorption by transporting glucose [12]. An abnormal expression of urate transporters leads to an increase in renal tubule reabsorption or a decrease in secretion, and consequently elevates blood uric acid, resulting in hyperuricemia and increased risk crease in gout attacks [13]. In this study, we found that relatively low concentrations of UA had no effect on the expression of URAT1 and GLUT9, but high concentrations of UA upregulated the expression levels of the two transporters. These results indicate that a high content of UA inhibits urate excretion from kidneys, resulting in more urate accumulation in blood and further development of hyperuricemia. On the other hand, FPS exposure reduced contents of the two transporters in HK-2 cells, suggesting that FPS successfully inhibited the expression induction of URAT1 and GLUT9 by UA. These in vitro findings supported the reported in vivo research results based on mice and rats [16,17,18,19].

It is documented that soluble uric acid induced the expression of GLUT9 and URAT1 in renal tubular epithelial cells of hyperuricemia mice/rats and was inhibited by FPS [16,17,18,19], which was reconfirmed by these in vitro findings, suggesting that FPS is effective in repressing reabsorption of urate from kidneys. The in vivo study by Chau et al. found that soluble uric acid also suppressed the expression of organic anion transporter 1 (OAT1) and human ATP-binding cassette (subfamily G2, ABCG2), two urate transporters that are responsible for urate transportation from blood to lumen, but FPS disinhibited the negative effect of UA on the two transporters’ expressions [19]. These data prove that FPS can improve urate excretion from kidneys, and alleviate hyperuricemia. Apart from renal excretion, intestinal excretion is another way for UA to enter blood—it is estimated that 10% blood’s uric acid is excreted from the intestines—so intestinal urate transporters are also important for blood urate depletion. Chen and co-workers found, based on HT-29 and Caco-2 human intestinal cell lines, that soluble uric acid promotes the expression of ABCG2, and improves intestinal excretion of uric acid through activating the PI3K/Akt pathway [20]. The result was opposite to that found in kidneys. Additionally, it is still unknown whether FPS can regulate the expression of intestinal ABCG2, and other urate transporters, in hyperuricemia animals. So, it cannot be deduced whether, or how, FPS promotes urate excretion from intestines.

It has been proven that UA can activate NF-κB signaling in proximal renal tubular epithelial cells and upregulate URAT1 expression [21]. Work from our laboratory also reported that adenine-induced hyperuricemia mice were found to have activated NF-κB signal pathways and increased URAT1 content in their kidneys [18]. This investigation re-observed the activation of the NF-κB signal pathway and the expression induction of URAT1 in high concentrations of UA-exposed HK-2 cells. The hyperuricosuria, caused by hyperuricemia and urate transporter upregulation, will probably increase the quantity of UA in proximal tubular cells, which will aggravate UA-induced tubule dysfunction [22]. Our early in vivo studies have confirmed that production inhibition and excretion promotion of urate by FPS in adenine-induced hyperuricemic mice and FPS inhibits NF-κB signal pathway activation via inhibiting phosphorylation of the NF-κB p65 subunit [18]. In this study, both QNZ and FPS inhibited NF-κB p65 phosphorylation induced by UA in HK-2 cells, suggesting that FPS played a role in inhibiting the UA-induced expression of URAT1 and GLUT9 through the NF-κB pathway.

It has been reported that MAPKs are pivotal mediators in the pathophysiology of kidney insults [23], and that JNK pathway is involved in the occurrence of UA-related diseases [24]. Li et al. found that monosodium urate crystal stimulation could activate the activities of NF-κB and MAPK pathways, and upregulate the phosphorylation of JNK in THP-1 cells in murine models of gout [25], which is similar to our results. In our study, UA activated the MAPK signaling pathway of HK-2 cells and enhanced the phosphorylation of JNK. However, when we combined the JNK inhibitor with UA to treat cells, the JNK signaling pathway activity was reduced. Furthermore, FPS reduced the activity of JNK signaling pathways conducted by UA, while FPS alone did not negatively affect the JNK MAPK signaling pathway. It is revealed that FPS may reduce UA damage to HK-2 cells via the JNK signaling pathway. Many studies have shown that FPS plays an anti-cancer role through the MAPK pathway [26,27].

Increasing evidence links hyperuricemia nephropathy to epithelial−mesenchymal transition (EMT), which is an essential process during renal fibrosis [28,29]. The PI3K/Akt pathway can interact with other pathways, including NF-κB and Wnt/catenin, to promote the development of EMT [30]. This study showed that UA promoted Akt activation. On the contrary, wortmannin could inhibit the effect of UA, which is consistent with the research of Xiong et al. [31]. Similarly, HK-2 cells were treated with FPS combined with UA, it was found that FPS could reverse the UA-associated effects of activation of Akt and increase in GLUT9 and URAT1 expression. FPS also protected HK-2 cells from UA damage through the PI3K/Akt pathway. In in vitro and in vivo studies of diabetic nephropathy, low-molecular-weight fucoidan significantly increased E-cadherin expression and reduced *α*-SMA, CTGF, and fibronectin expression in both type 1 and type 2 diabetic models. It also decreased the phosphorylation of Akt, ERK1/2, p38 and Smad3, thereby protecting kidneys from dysfunction and fibrogenesis by inhibiting the TGF-*β* pathway [32].

## 4. Materials and Methods

### 4.1. Cells Culture

HK-2 cells purchased from ATCC and were grown in an Roswell Park Memorial Institute (RPMI) 1640 medium, supplemented with 10% heat-inactivated foetal bovine serum (FBS, Gibco, Grand Island, NY, USA) and 1% penicillin-streptomycin, at 37 °C in an incubator containing 5% CO_2_ atmosphere. The medium was changed every 2–3 days, and cells were subcultured at 80% confluence for the following work.

### 4.2. Western Blotting Analysis

Cells were washed with PBS, and were then lysed in RIPA buffer containing PMSF (Sigma, St. Louis, MO, USA) at 4 °C. The lysate was centrifuged at 12,000× *g* rpm for 5 min at 4 °C. The supernatant was detected as the protein content, using a BCA protein assay kit, and was then diluted in a protein loading buffer followed by heat inactivation in a boiling water bath for 10 min. Samples were subjected to sodium dodecyl sulfate polyacrylamide gel electrophoresis, followed by transferal to polyvinylidene fluoride (PVDF) membranes (Millipore, Billerica, MA, USA). After being treated in 5% skim milk in TBST for 1 h, the membranes were probed overnight at 4 °C with the primary antibodies against URAT1, GLUT9, p65, *p*-p65, JNK, *p*-JNK, AKT, *p*-AKT or *β*-actin (Abcam, Cambridge, UK or Proteintech, Rosemont, IL, USA), respectively. Following washing with Tris-buffered saline Tween 20 (TBST) three times, the membranes were transferred into a corresponding secondary antibody solution, and incubated for 1 h at room temperature. Afterwards, the membranes were washed three times with TBST and were detected on a multifunction imager by using the enhanced chemiluminescence (ECL) kit. The intensity of individual bands were normalized to *β*-actin. Value was expressed as the ratio of treated group to vehicle group.

### 4.3. Immunofluorescent (IF) Staining

Cells were washed with PBS, and were then fixed with 4% paraformaldehyde for 20 min at room temperature. After washing with PBS, cells were permeabilized with 0.1% Triton X-100 for 15 min at room temperature. HK-2 cells were washed with PBS and were incubated in blocking buffer for 2 h at room temperature. Cells were probed at 4 °C overnight with the primary antibody against URAT1 or GLUT9. Cells washed with PBS were incubated with an FITC-conjugated secondary antibody for 1 h at room temperature. Following washing with PBS, Hoechst 33342 was used to stain nuclei for 20 min at room temperature. After washing with PBS, fluorescence was examined with a fluorescence microscope, Ts2R-FL (Nikon, Tokyo, Japan).

### 4.4. Statistical Analysis

The data were shown as average and standard deviation. SPSS 22.0 software was used for statistical analysis. One-way ANOVA and LSD were used to make multiple comparisons for evaluating the statistical significance. *p* < 0.05 was taken as statistical significance.

## 5. Conclusions

A relatively higher concentration of UA can promote the expression of GLUT9 and URAT1 via activating the NF-κB, JNK and PI3K/Akt signaling pathways in renal tubular epithelial cells. However, UA caused activation of the pathways can be inhibited by FPS, resulting in expression downregulation of the two urate reabsorption transporters. These results support the reported in vivo research findings, and prove that FPS inhibits the expression of GLUT9 and URAT1 in renal tubular epithelial cells via inhibiting the UA-associated activation of the NF-κB, JNK and PI3K/Akt signaling pathways to promote urate excretion from kidneys, consequently decreasing blood uric acid content in hyperuricemia mice. It is interesting that the bio-function of FPS only occurs in UA-exposed cells, not in control cells. These results indicate that, as a clinical nephropathy drug which has been used for nearly 20 years in China, FPS should be a potential marine drug for hyperuricemia and gout.

## Data Availability

Data are contained within the article.
